# A Balance between Nuclear and Cytoplasmic Volumes Controls Spindle Length

**DOI:** 10.1371/journal.pone.0149535

**Published:** 2016-02-17

**Authors:** Lucia Novakova, Kristina Kovacovicova, Thanh Quang Dang-Nguyen, Martin Sodek, Michal Skultety, Martin Anger

**Affiliations:** 1 Central European Institute of Technology - Veterinary Research Institute, Hudcova 70, 621 00 Brno, Czech Republic; 2 Institute of Animal Physiology and Genetics AS CR, Rumburska 89, 277 21 Libechov, Czech Republic; Virginia Tech, UNITED STATES

## Abstract

Proper assembly of the spindle apparatus is crucially important for faithful chromosome segregation during anaphase. Thanks to the effort over the last decades, we have very detailed information about many events leading to spindle assembly and chromosome segregation, however we still do not understand certain aspects, including, for example, spindle length control. When tight regulation of spindle size is lost, chromosome segregation errors emerge. Currently, there are several hypotheses trying to explain the molecular mechanism of spindle length control. The number of kinetochores, activity of molecular rulers, intracellular gradients, cell size, limiting spindle components, and the balance of the spindle forces seem to contribute to spindle size regulation, however some of these mechanisms are likely specific to a particular cell type. In search for a general regulatory mechanism, in our study we focused on the role of cell size and nuclear to cytoplasmic ratio in this process. To this end, we used relatively large cells isolated from 2-cell mouse embryos. Our results showed that the spindle size upper limit is not reached in these cells and suggest that accurate control of spindle length requires balanced ratio between nuclear and cytoplasmic volumes.

## Introduction

Faithful chromosome segregation is vital for transfer of intact genetic information into the daughter cells. A central role in this process is played by the spindle microtubule apparatus, which is involved in all crucial steps of chromosome division [1,2]. From engagement of the chromosomes into division by capturing kinetochores during the early stages of mitosis, through their alignment at the metaphase plate, up to the distribution of sister chromatids into daughter cells during the final stages of mitosis, all these functions are mechanistically carried out by the spindle. Although proper assembly of the spindle is required for accurate chromosome segregation, our understanding of the molecular mechanisms controlling this process is still incomplete. A clear example is the regulation of spindle length, which is important for faithful chromosome segregation [3], as well as for asymmetric cell division [4]. Although it seems that in general spindle length is predetermined by the cell size, in certain cells, such as early mammalian embryos, it seems that the length of the spindle is regulated, to some extent, independent of cell size [5–8]. Similarly, in *Xenopus* embryos during initial cleavage cycles, the reduction in spindle size is not proportional to the decrease in cell size [9]. In these gigantic cells, spindle size is not proportional to the cell diameter and it is regulated by an upper limit, whereas when cells become smaller later in development, spindle size is more and more controlled by cell size [9–11]. Various mechanisms were shown to contribute to the regulation of spindle length. These include molecular gradients [12–14], density of kinetochore-microtubule attachments [15], a balance of the spindle forming forces [1], and limited availability of spindle building blocks in the cytoplasm [10,11]. In this study, we focused on a role of cell volume and nuclear to cytoplasmic ratio in the regulation of spindle length in mammalian blastomeres from 2-cell stage mouse embryos. By manipulating cell volume and nuclear to cytoplasmic ratio in combination with live cell imaging we discovered that cell size as well as the nuclear to cytoplasmic ratio have significant effect on spindle length. This indicates that the blastomeres of early cleavage cycles of mouse embryos regulate their spindle size by cell volume and by balanced equilibrium between nuclear and cytoplasmic volumes.

## Materials and Methods

### Animals

All animal work was conducted according to Act No 246/1992 Coll., on the protection of animals against cruelty and was approved by the Central Commission for Animal Welfare, approval ID 1505/2013 and 1566/2014 and supervised by the local institutional Expert committee for ensuring welfare of experimental animals of Veterinary Research Institute in Brno (*Odborná komise pro zajišťování dobrých životních podmínek pokusných zvířat*). All experiments were carried out following the rules of reduction of numbers of animals and minimizing their suffering during the experiments. BDF1 male mice were purchased from Anlab, Czech Republic. ICR/BDF1 female mice were obtained from crossing between ICR female (Animal Breeding and Experimental Facility, Faculty of Medicine, Masaryk University, Czech Republic) and BDF1 male (Anlab, Czech Republic). Experiments were performed using adult, 10–18 week old, animals.

### Mouse stimulation, embryo handling

ICR/BDF1 mice were stimulated with pregnant mares serum gonadotropin (PMSG, 5 IU, Sigma Aldrich, Czech Republic) and human chorionic gonadotropin (hCG, 5 IU, Sigma Aldrich, Czech Republic) at a 44–48 hour interval. To collect embryos, mice were mated at the time of hCG administration. MII oocytes were collected 14–16 h after hCG administration from non-mated mice, 2-cell embryos were collected 40–44 h after hCG administration. MII oocytes and embryos were collect by manual rupturing of the ampulla. The cumulus cells were removed from MII oocytes by pipetting in M2 medium supplemented with hyaluronidase (150 IU/ml, Sigma Aldrich, Czech Republic). Cells were subsequently cultured in KSOM AA medium (Caisson, USA), covered with mineral oil at 37°C, 5% CO_2_.

### Microinjection and micromanipulations

Microinjection was performed in M2 medium (Sigma Aldrich, Czech Republic) with 1–10 microinjector (Narishige) on a Leica DM IL inverted microscope. Complementary RNAs for microinjection were prepared by *in vitro* transcription (mMESSAGE mMACHINE and Poly(A) Tailing kit, Lifetechnologies, Czech Republic) of plasmids containing ORFs of mouse β-tubulin, TPX2, and H2B as transcription fusion with the sequence encoding fluorescent proteins EGFP, Venus, and mCherry, respectively. Enucleation was performed on a Leica DMI3000 B inverted microscope equipped with Eppendorf InjectMan^®^ NI 2 Micromanipulator (Eppendorf, Czech Republic). 2-cell embryos were transferred into M2 medium supplemented by cytochalasin B (Sigma Aldrich, Czech Republic) for at least 15 minutes prior to micromanipulation. The nucleus was removed from one blastomere of the 2-cell embryo using a piezo drill-assisted micromanipulation system with a 12-μm-diameter pipette. The zona pellucida was removed from 2-cell embryos by treatment with 1% pronase (Sigma Aldrich, Czech Republic) dissolved in M2 medium. Blastomeres of 2-cell embryo were separated manually in M2 medium. 2-cell embryos or 3 separated blastomeres from 2-cell embryo were transferred into 0.3 mg/ml phytohemagglutinin (Sigma Aldrich, Czech Republic) in M2 medium for 30 minutes prior to fusion. The fusion of agglutinated cells was performed in 1mm fusion chamber with two direct current pulses of 75V for 50 μsec (2-cell embryo) or with 2 single pulse of 50V for 40 μsec (3 blastomeres) using Multiporator (Eppendorf, Czech Republic).

### Parthenogenetic activation

MII oocytes were parthenogenetically activated by 4.5 min cultivation in 7% ethanol in M2 medium. After activation cells were transferred into KSOM AA medium (Caisson, USA), covered with mineral oil and cultured at 37°C, 5% CO_2_ for 6–7 hours, after which cells were scored for pronuclei and microinjected. Embryos were transferred to microscope for live imaging at the 2-cell stage, approx. 47 hours after activation.

### Live cell imaging

Embryos were transferred to a Leica SP5 confocal microscope, equipped with an EMBL incubator allowing time-lapse experiments in 5% CO2 at 37°C. 488, 514, and 561 nm excitation wavelengths, HCX PL APO 40 x /1.1 water objective, and hybrid detectors were used for detection of EGFP, Venus and mCherry fluorescent proteins. Z-stacks of 31 or 41, depending on the cell size, images were taken every 10 minutes.

### Image analysis

Image analysis, spindle measurement, and nucleus and cytoplasm volume measurements were performed using Imaris software 7.6.5 (Bitplane AG, Switzerland, www.bitplane.com). Statistical analysis was performed using Prism software, version 5.00 for Mac (GraphPad Software, San Diego California USA, www.graphpad.com). Mean and SD values were calculated using MS Excel (Microsoft). The statistical significance of the difference between control and experimental group was tested using t-test or ANOVA.

## Results

### Spindle length scales with cell volume

**Fig 1:**
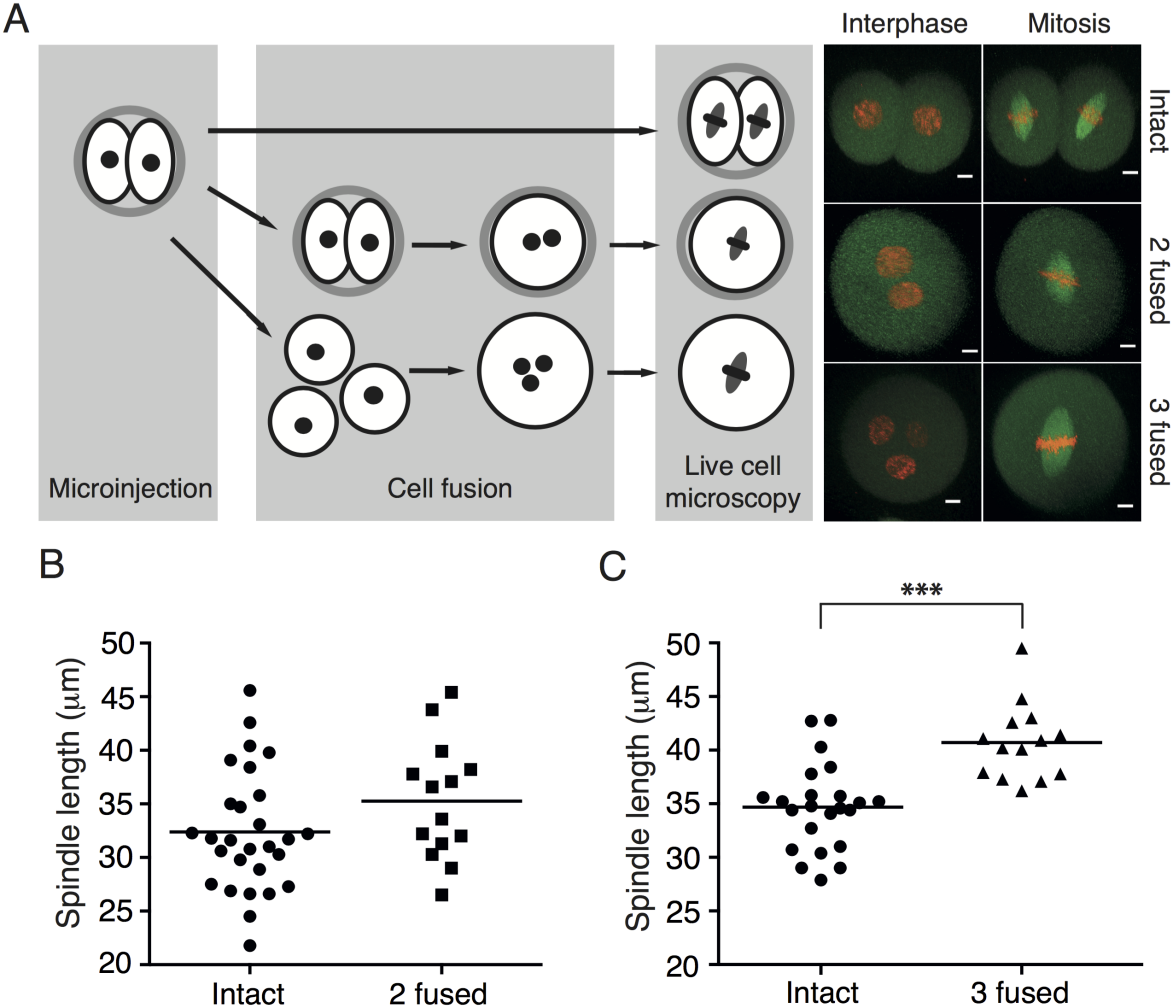
Spindle length increases proportionally with cell volume. (A) Left panel: Blastomeres of mouse 2-cell embryos were microinjected with cRNAs encoding Histone-H2B fused to mCherry and Tubulin fused to EGFP. The following cells were used for experiments: intact two cell embryos (Intact), cells with double volume obtained by fusion of two blastomeres (2 fused), and cells with triple volume obtained by fusion of three blastomeres (3 fused). Right panel shows representative movie frames from time lapse imaging of each cell type in interphase and in mitosis; chromosomes are in red, spindle is in green, scale bar represents 10 μm. (B) The length of the spindle in 2 fused cells (35.26 ± 5.52 μm, n = 14) was not significantly increased (p = 0.1236) in comparison to the length of the spindle in Intact cells (32.38 ± 5.63 μm, n = 28). (C) The length of the spindle in 3 fused cells (40.71 ± 3.57 μm, n = 14) was significantly increased (p < 0.0001) in comparison to the length of the spindle in Intact cells (34.68 ± 3.99 μm, n = 23).

In order to study the relationship between cell volume and spindle length, we employed a challenging procedure, during which two or three blastomeres from 2-cell embryos were electrofused, giving rise to cells with double or triple volume compared to an intact blastomere. The following cells were created and used in our experiments: intact blastomeres from 2-cell embryos (Intact), two fused blastomeres from 2-cell embryos (2 fused), and three fused blastomeres from 2-cell embryos (3 fused) (Fig 1A, left panel). Prior to the fusion, all cells were microinjected with cRNAs encoding Histone H2B and Tubulin tagged with fluorescent proteins; this was important for monitoring chromosome division and for measuring spindle size during the subsequent mitosis. It was shown previously that the duration of the second mitosis in mouse embryos is approximately 70 minutes [16]. We decided to avoid pharmacological synchronization in order to obtain spindles with unperturbed structure and function. However, under these conditions, the time interval during which cells are in metaphase is relatively narrow. To circumvent this, we used confocal live cell imaging and recorded the entire cell division. Cells were cultured on a confocal microscope equipped with temperature and CO_2_ control and z-stacks of 31 or 41 images were taken every 10 minutes (Fig 1A, right panel). Our settings allowed good coverage of the whole cell volume and therefore morphological parameters of the spindles were possible to measure for each cell. The size of the spindle and the cell was measured using image stacks acquired during the last time interval before anaphase. Our results (Fig 1B) showed that the average spindle length in Intact cells was 32.38 ± 5.63 μm. The spindle size in 2 fused cells was increased, although not significantly, in comparison to the intact cells, on average it was 35.26 ± 5.52 μm. However, the spindles in 3 cells fused were significantly longer, measuring 40.71 ± 3.57 μm (Fig 1C). Although the spindle size difference between intact and 2 fused cells exists, only in 3 fused cells the difference in cell volume was sufficiently big to detect the variations in spindle size within a relatively narrow cohort of cells. In 3 fused cells the spindles were not only longer, but they were also significantly wider, which was probably due to a larger number of chromosomes occupying the equatorial plane (S1 Fig).

### Metaphase spindle length is regulated by a balanced ratio between nuclear and cytoplasmic volumes

**Fig 2:**
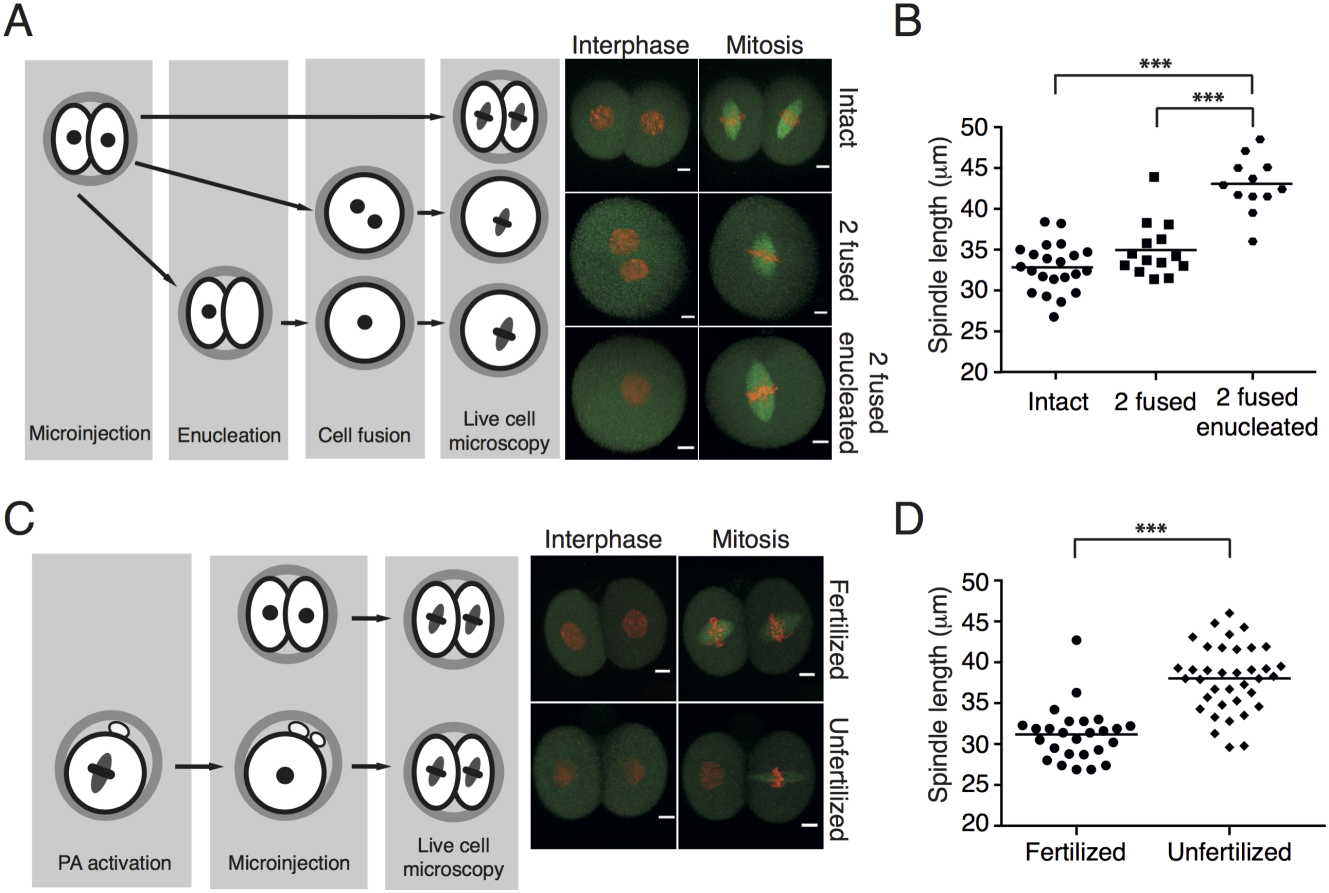
Changes in nuclear to cytoplasmic ratio have impact on spindle length. (A) Left panel: Blastomeres of mouse 2-cell embryos were microinjected with cRNAs encoding Histone-H2B fused to mCherry and Tubulin fused to EGFP. The following cells were used for experiments: intact two cell embryos (Intact), cells with double volume obtained by fusion of two blastomeres (2 fused), and cells with double volume of the cytoplasm and impaired nuclear to cytoplasmic ratio resulting from enucleation (2 fused enucleated). Right panel shows representative movie frames from time lapse imaging of each cell type in interphase and in mitosis; chromosomes are in red, spindle is in green, scale bar represents 10 μm. (B) The length of the spindle in 2 fused enucleated cells (42.91 ± 3.35 μm, n = 12) was significantly increased (p < 0.0001) in comparison to the length of the spindle in intact cells (32.83 ± 2.95 μm, n = 22) or 2 fused cells (34.96 ± 3.37 μm, n = 14). (C) Left panel: haploid embryos were produced by parthenogenetic activation of metaphase II eggs. Following activation, cells were microinjected with cRNAs encoding Histone-H2B fused to mCherry and Tubulin fused to EGFP together with blastomeres of 2-cell embryos. Right panel shows representative movie frames from time lapse imaging of each cell type in interphase and in mitosis; chromosomes are in red, spindle is in green, scale bar is 10 μm. (D) The length of the spindle in Unfertilized haploid cells (38.04 ± 4.06 μm, n = 37) was significantly increased (p < 0.0001) in comparison to the length of the spindle in intact cells (Fertilized) (31.18 ± 3.32 μm, n = 26).

**Fig 3:**
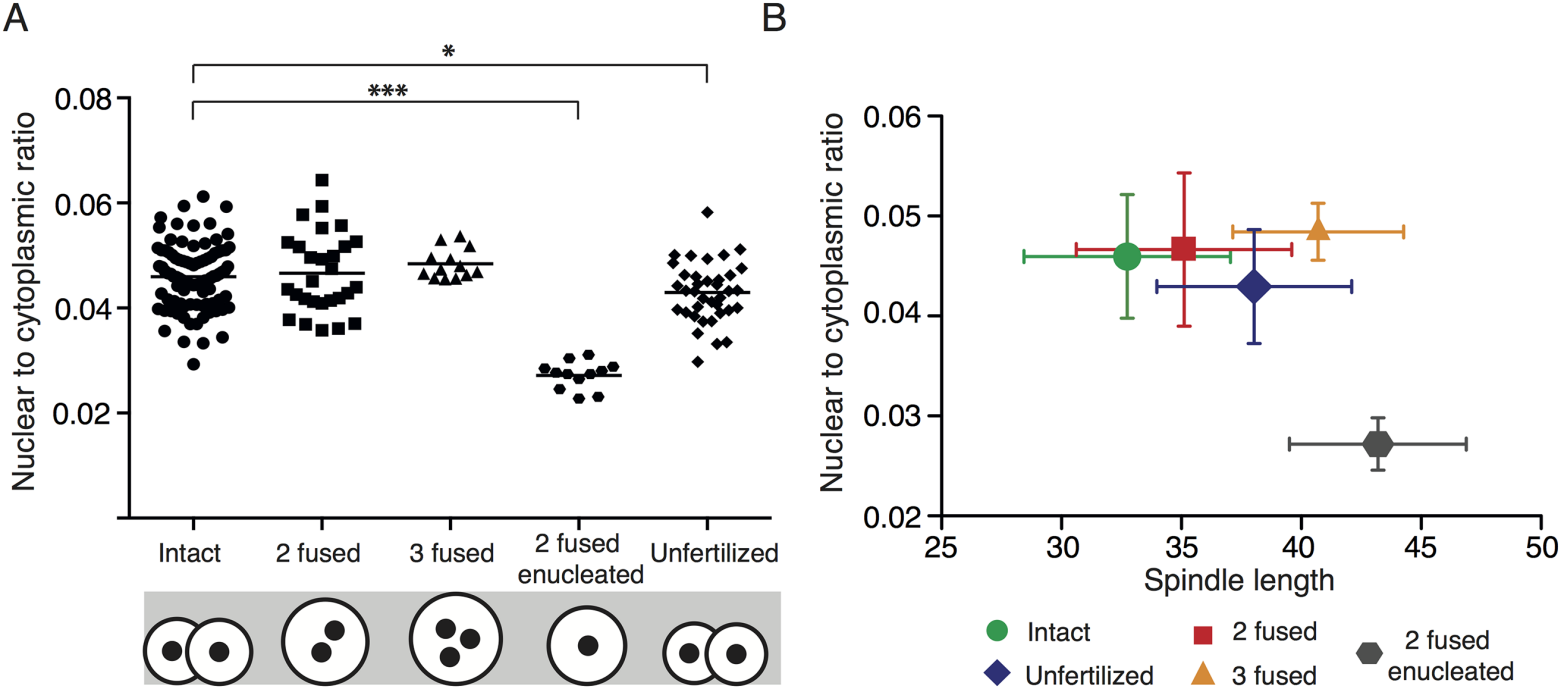
Nuclear to cytoplasmic ratio in cells created by fusion, enucleation, or parthenogenetic activation. Panel A: The chart shows nuclear to cytoplasmic ratio in all cells used in experiments described in Figs 1 and 2: Intact cells (0.046, n = 93), 2 fused cells (0.047, n = 28), 3 fused cells (0.048, n = 13), 2 fused enucleated cells (0.027, n = 12) and unfertilized cells (0.043, n = 40). N:C ratio of Intact cells is significantly different in comparison to 2 fused enucleated (p < 0.0001) and unfertilized cells (p = 0.0116). The diagram below the X-axis illustrates the proportional differences between cell and nuclear size of each cell type. Panel B: The plot shows a relationship between nuclear to cytoplasmic ratio and spindle length in all cells used in experiments described in Figs 1 and 2: Green dots represent intact cells (spindle length: 32.75 ± 4.30; NC ratio: 0.046 ± 0.006), red squares represent 2 fused cells (spindle length: 35.11 ± 4.49; NC ratio: 0.047 ± 0.008), orange triangles represent 3 fused cells (spindle length: 40.70 ± 3.56; NC ratio: 0.048 ± 0.003), blue rhombuses represent unfertilized cells (spindle length: 38.04 ± 4.06; NC ratio: 0.043 ± 0.006) and gray hexagons represent 2 fused enucleated cells (spindle length: 43.20 ± 3.69; NC ratio: 0.027 ± 0.003).

**Fig 4:**
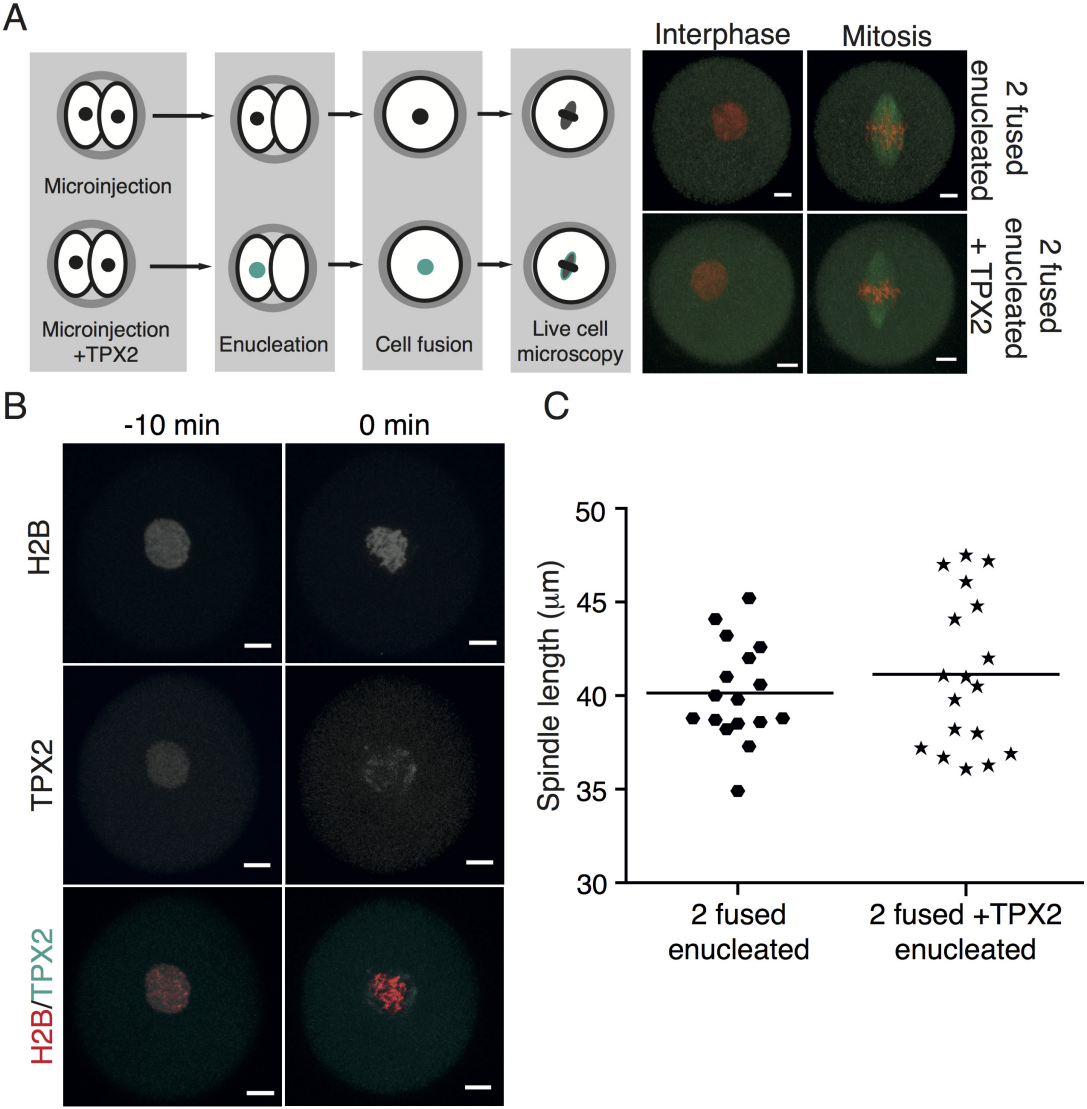
Overexpression of TPX2 has no effect on spindle length. (A) Left panel: Blastomeres of mouse 2-cell embryos were microinjected with cRNAs encoding Histone-H2B fused to mCherry and Tubulin fused to EGFP with (bottom) or without (top) cRNA encoding TPX2 protein. Following microinjection, one of the two blastomeres in each embryo was enucleated and then both fused together. Right panel shows representative movie frames from time lapse imaging of each cell type in interphase and in mitosis, chromosomes are in red, spindle is in green, scale bar represents 10 μm. (B) In some experiments, TXP2 was used with fluorescent tag. Representative images of cells before (left panels, 10 min) and during NEBD (right panel, 0 min) show the nuclear localization of exogenous TPX2 in interphase and its relocalization to the cytoplasm before spindle assembly. Chromosomes are in red, TPX2 in cyan, scale bar represents 10 μm. (C) The length of the spindle in 2 fused enucleated cells with overexpressed TPX2 (41.14 ± 4.07 μm, n = 18) was not significantly different (p = 0.3961) compared to the length of the spindle in 2 fused enucleated cells (40.14 ± 2.64 μm, n = 17).

It was suggested that the number of chromosomes or kinetochores affects spindle length [15,17]. To test this, we compared spindle size in intact cells with 40 chromosomes (Intact) with spindle size in 2 fused cells with 80 chromosomes and two fused cells in which one of them was enucleated before fusion (2 fused enucleated) and therefore carried 40 chromosomes (Fig 2A). As in previous experiments, all cells were microinjected with cRNAs encoding Histone and Tubulin fusion proteins in order to follow chromosomes and spindles during cell division. The difference in spindle length between intact (32.83 ± 2.95 μm) and 2 fused cells (34.96 ± 3.37 μm) was not statistically significant, similarly to our previous experiments. However, the difference between these two groups and 2 fused enucleated cells was dramatic, with spindles measuring on average 42.91 ± 3.35 μm, in the latter group (Fig 2B). We also measured the width of the spindle in all three groups (S2 Fig). The spindle was wider in 2 fused than in intact cells or 2 fused enucleated cells. Since Intact and 2 fused enucleated cells harbor the same number of chromosomes, it seems that spindle width in this cell type is controlled by the number of chromosomes. Our results indicated that the number of chromosomes/kinetochores does not control spindle length since the Intact and 2 fused enucleated cells contain the same number of chromosomes/kinetochores although their spindle size differs significantly. To confirm this, we employed a different method for reducing chromosome numbers and prepared 2-cell blastomeres from parthenogenetically activated metaphase II eggs (Unfertilized) and compared them to intact blastomeres of 2-cell embryos (Fertilized) (Fig 2C). Spindle length in unfertilized haploid embryos was significantly increased (38.04 ± 4.06 μm) in comparison to intact control cells (31.18 ± 3.32 μm) (Fig 2D), whereas spindle width was greater in fertilized cells, which contained more chromosomes (S3 Fig). Therefore both cell types in which the number of chromosomes was reduced, namely 2 fused enucleated and Unfertilized cells, showed significant increase of mitotic spindle length. We were however puzzled by other cell types, which we created and in which the changes in chromosome numbers did not show this effect. Intact diploid blastomeres carrying 40 chromosomes with spindle length 32.65 ± 4.20 μm (average from all experiments), 2 fused cells carrying 80 chromosomes with spindle length 37.62 ± 6.49 μm, 3 fused cells with 120 chromosomes and spindle length 40.15 ± 2.61 μm, 2 fused enucleated cells with 40 chromosomes and spindles 41.54 ± 3.15 long, and finally parthenogenetically activated cells with 20 chromosomes and spindle length 38.04 ± 4.06 μm. From this comparison, it seems very likely, that the number of chromosomes may not be a major factor controlling spindle length. In order to search for other potential differences between these cells, we measured the nuclear to cytoplasmic (N:C) ratio in the last frame before NEBD (Fig 3A). It revealed that the two groups with significantly lower N:C ratio in comparison to intact cells are 2 fused enucleated and unfertilized cells. Further comparison of N:C ratio and spindle length showed that cells with significantly lower N:C ratio had longer spindles (Fig 3B). This correlation was specifically apparent in 2 fused enucleated cells, which had the lowest N:C ratio and also the longest spindles. We concluded that maintenance of a balanced ratio between cytoplasm and nucleus is important for accurate regulation of spindle length, whereas the number of chromosomes is important for spindle width. We believe that the simplest explanation of our experiments would envisage unknown component or components are lower in cells in which the nuclei are smaller compared to the cell volume. Several recent studies showed that the activity of spindle assembly factor TPX2 is important for the regulation of spindle length [18–20]. Because of its exclusive nuclear localization before cell division, this was a good candidate as the decreased component caused by the relatively smaller nucleus vs. the volume of the cytoplasm. We tested the effect of TPX2 overexpression on spindle length using 2 fused enucleated cells, which displayed the longest spindles. Cells were microinjected with cRNAs encoding Histone and Tubulin fused to fluorescent proteins and half of the cells also with cRNA encoding TPX2 (Fig 4A). In some experiments the TPX2 was also fused to fluorescent protein in order to assess its localization during interphase and in mitosis. The localization of TPX2 in 2 fused enucleated cells was exclusively nuclear in interphase with transition to the whole cell volume as cells entered mitosis (Fig 4B).The overexpression of TPX2 in concentrations preserving intact spindles did not significantly affected spindle size (Fig 4C) and therefore we concluded that this factor is not crucial for the regulation of spindle length in this cell type.

## Discussion

One set of fundamental questions in cell biology, which still remains to be elucidated, is how the size of organelles and intracellular structures is regulated [21]. Such regulation must be able to flexibly respond to dramatic changes, emerging for example during embryonic development, which is characterized by rapid sequential cell cycles with substantial decrease of the cell volume during every cleavage. In our study, we focused on general rules regulating spindle size, namely the role of cell size, nuclear to cytoplasmic ratio, and number of chromosomes in this process. We employed large cells of mouse 2-cell embryos because due to their size the changes of the cell volume or nuclear to cytoplasmic ratio can be achieved. Our cell fusion experiments showed that the size of the spindle in early mammalian embryos is controlled by the cell size. This is in contrast with data obtained using *Xenopus* embryos, where the spindle size in initially extremely large embryonic cells has a maximum size limit [9]. Our cell fusion experiments suggested that the upper limit of the spindle is either not active in size regime of mouse embryo or was not reached in our experiments. It was shown that during mouse early embryonic cell cleavages spindle size decreases more slowly than cell size [5,8]. Also, removal of a substantial amount of the cytoplasm did not cause shortening of the spindle [5], suggesting that spindle size during this developmental period is at its lower limit. It was shown that the size of the organelles and subcellular structures is controlled by the pool of the available cytoplasmic components (reviewed in [22]). In *C*. *elegans* embryo for example, spindle length is regulated by centrosome size [13] and centrosome size is in turn regulated by limited amount of centrosome material in the cytoplasm [23]. Recently published work using *Xenopus* cell free extracts showed that in this system spindle size is controlled by the amount of cytoplasm [10,11]. To test this hypothesis using our model system, we compared cells which resulted from fusion of two equal 2-cell embryo blastomeres or one intact and one enucleated blastomere, therefore we obtained cells with different N:C ratio. If the size of the spindle is controlled by the availability of cytoplasmic components, in both groups the spindle should be equally increased. However, the spindles in 2 fused enucleated cells were significantly longer indicating that the cytoplasmic components are not limiting for the spindle size in these cells. Those results, in our opinion indicate that rather than by the amount of cytoplasm, spindle size is controlled by N:C ratio (Fig 3). The N:C ratio was shown to participate in regulation of zygotic gene activation at the midblastula transition in *Xenopus* [24]. The changes in this ratio influence a variety of cell behaviors [25,26] and it is conceivable that they could also have an effect on the regulation of spindle length. However, we need to keep in mind that our experimental procedure is not directly comparable to N:C changes during early development and the changes may not be limited to the N:C ratio. Indeed, it was recently shown that in Xenopus oocytes many proteins are exclusively located either in the nucleus or in the cytoplasm [27]. Therefore, during enucleation, proteins within nucleus are specifically depleted, which might change the stoichiometry of the system. In conclusion, our study brings some new insight into regulation of spindle length in early mouse embryo, namely the role of the nuclear to cytoplasmic ratio in this process. However, the molecular basis of this regulation needs to be revealed by future work. Obtaining more information about mechanisms controlling chromosome segregation, such as for example regulation of spindle length, is vital since mammalian early embryos are highly aneuploid [28] and it is conceivable that failure of the mechanisms controlling spindle size might contribute to the increased frequency of chromosome segregation errors.

## Supporting Information

S1 Fig The spindle of 3 fused embryos (21.27 ± 1.32 μm, n = 13) is significantly (p < 0,0001) wider in comparison to spindle of Intact cell (13.14 ± 0.72 μm, n = 21). (TIFF)

S2 Fig The spindle of 2 fused embryos (17.29 ± 1.01 μm, n = 14) is significantly wider (p < 0.0001) than the spindle of 2 fused embryos (15.28 ± 1.17 μm, n = 12) and intact embryos (13.17 ± 1.04 μm, n = 22). (TIFF)

S3 Fig The spindle of fertilized embryos (13.21 ± 1.14 μm, n = 26) is significantly wider (p < 0,0001) compared to the spindle of unfertilized embryos (9.42 ± 1.47 μm, n = 37). (TIFF)
