# Supplementary figures and images for: A Balance between Nuclear and Cytoplasmic Volumes Controls Spindle Length
Source: PLoS One. 2016 Feb 17;11(2):e0149535. doi: 10.1371/journal.pone.0149535 (PMC4757572; doi:10.1371/journal.pone.0149535)

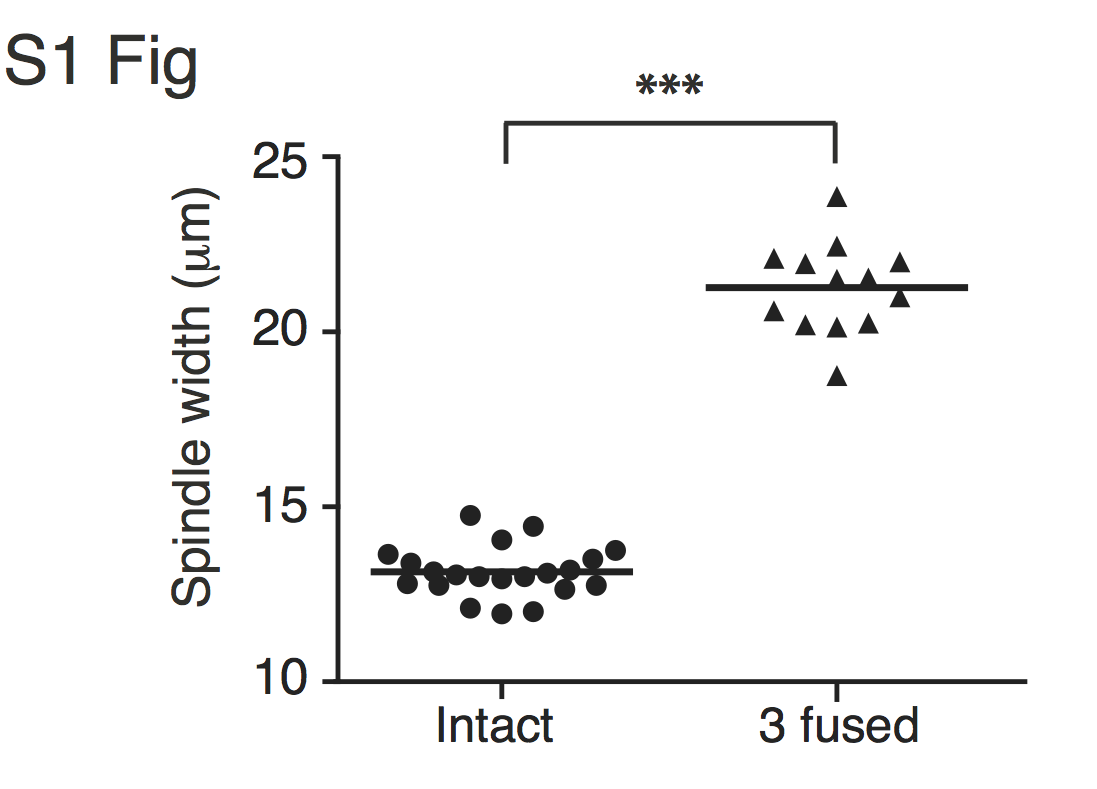

Supplement: S1 Fig — (TIFF) [file pone.0149535.s001.tiff]

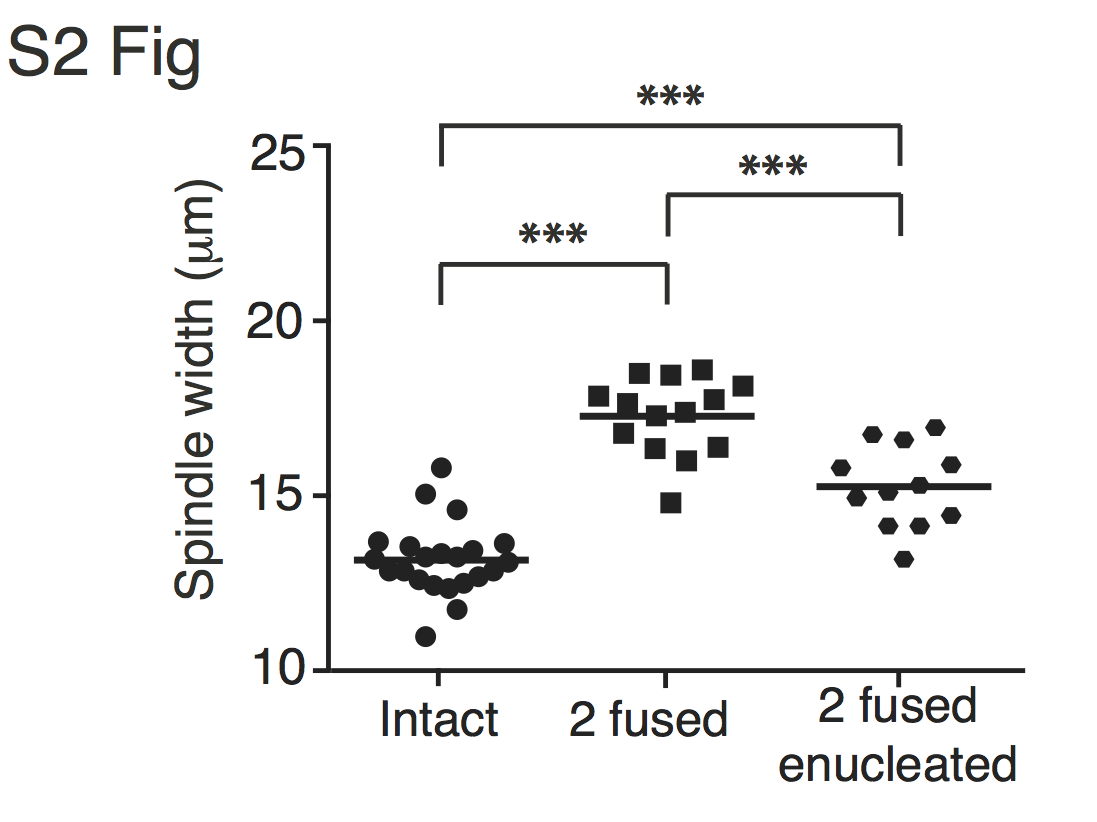

Supplement: S2 Fig — (TIFF) [file pone.0149535.s002.tiff]

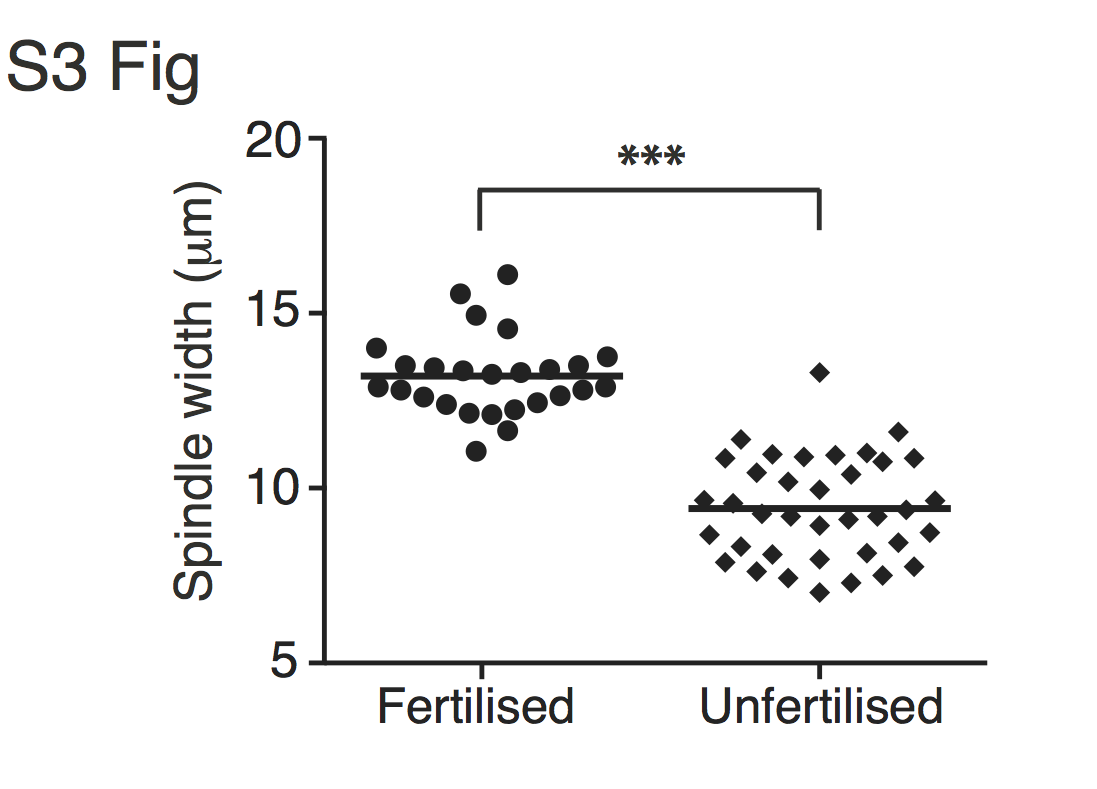

Supplement: S3 Fig — (TIFF) [file pone.0149535.s003.tiff]
